# Modeling symptom-acupoint interactions via a heterogeneous graph learning framework for intelligent acupoint recommendation

**DOI:** 10.1186/s13020-026-01458-1

**Published:** 2026-07-16

**Authors:** Wei Lin, Jiaqi Chen, Zhuo Chen, Tao Yin, Jing Guo, Fang Zeng

**Affiliations:** 1 School of Acupuncture, Moxibustion and Tuina, Chengdu University of Traditional Chinese Medicine, Chengdu, 610075 China; 2 Key Laboratory of Acupuncture for Senile Disease (Chengdu University of Traditional Chinese Medicine), Ministry of Education, Chengdu, 610075 China; 3 Key Laboratory of Acupuncture for Medicine Research (Nanjing University of Traditional Chinese Medicine), Ministry of Education, Nanjing, 210023 China; 4 School of Intelligent Medicine, Chengdu University of Traditional Chinese Medicine, Chengdu, 611137 China

**Keywords:** Acupuncture, Graph neural networks, Multi-label text classification, Functional gastrointestinal diseases

## Abstract

**Objective:**

Acupuncture prescriptions involve complex compatibility mechanisms grounded in multi-symptom and multi-acupoint interactions, embodying millennia of clinical experience. Despite growing interest in computational acupoint recommendations, significant challenges persist due to sparse clinical data and the insufficient modelling of symptom-acupoint relationships, posing considerable hurdles to effective prediction.

**Methods:**

We introduced an acupoint compatibility prediction framework with graph neural networks (GNN) and fine-tuned bidirectional encoder representations from transformers (termed GNN-BERT-Attention). The heterogeneous feature interaction learning mechanism was introduced to model symptom-acupoint interactions through heterogeneous graph construction, capturing semantic features and relational patterns in a unified space, which alleviated the sparsity of data. Neural collaborative filtering is utilised via label-aware fusion to iteratively refine the confidence of predictions, while Focal Loss and randomised augmentation strategies enhance robustness against imbalanced label distribution.

**Results:**

Comprehensive experiments demonstrate the superiority of the proposed GNN-BERT-Attention model over State-of-the-Art (SOTA) baselines in precision, recall, ranking-based metrics and robustness. Ablation studies validate the effectiveness of each architectural module, and hyperparameter tuning confirms models’ stability. A web-based demonstration system further validates clinical applicability, enabling real-time, interpretable acupoint recommendations.

**Conclusion:**

This study contributes to enhancing the performance of acupoint prediction, ultimately benefiting the efficiency and precision of acupuncture treatment while providing a theoretical foundation for optimising prescriptions and advancing evidence-based traditional Chinese medicine interventions.

## Introduction

Functional gastrointestinal disorders (FGIDs) are common chronic and recurrent psychosomatic conditions characterised by gastrointestinal symptoms such as heartburn, stomach pain, diarrhoea and bloating without identifiable organic causes [1]. The most common FGIDs include irritable bowel syndrome, functional dyspepsia and functional constipation affecting up to one–third of the FGIDs population [2]. These disorders significantly reduce patients’ quality of life and impose a substantial burden on society and healthcare systems [3, 4]. Acupuncture is widely used in FGIDs as a complementary and alternative therapy [5]. Numerous clinical studies have demonstrated that acupuncture can significantly improve the symptoms of FGIDs, reduce anxiety and depression in patients and enhance their overall quality of life [6]. However, there is no standardised protocol for acupoint selection, as each acupoint possesses multiple therapeutic functions. Traditional Chinese medicine (TCM) physicians collect patient symptoms through four diagnostic methods, including inspection, auscultation and olfaction, enquiry and palpation [7], then classify diseases into syndrome patterns based on the symptoms [8]. Each syndrome corresponds to a distinct set of key acupoints, resulting in a complex relationship between acupoints and diseases [9, 10].

Artificial intelligence excels at multidimensional data processing and modelling intricate relationships, thereby providing a new opportunity for investigating the complex mechanisms underlying acupuncture prescriptions [11]. Recent studies have explored a range of computational approaches for TCM acupoint recommendations. Knowledge graph-based methods, such as AcuKG [12], integrate heterogeneous acupuncture data into structured representations. Rule-based systems encode TCM diagnostic logic but suffer from limited scalability [13], particularly for complex historical texts. Meanwhile, traditional machine learning techniques [14] can identify frequent acupoint combinations from clinical data. However, these methods generally fall short of capturing rich semantic information in symptom descriptions and modelling higher-order interactions among acupoints.

Multi-label text classification (MLTC), as a significant task in natural language processing (NLP), can assign multiple labels or categories to each input text [15, 16]. Deep learning models, especially bidirectional encoder representations from transformers (BERT) and graph neural networks (GNN), can capture key information in the text and improve classification, thus having an advantage in handling text features and label relationships [17, 18].

**Fig. 1:**
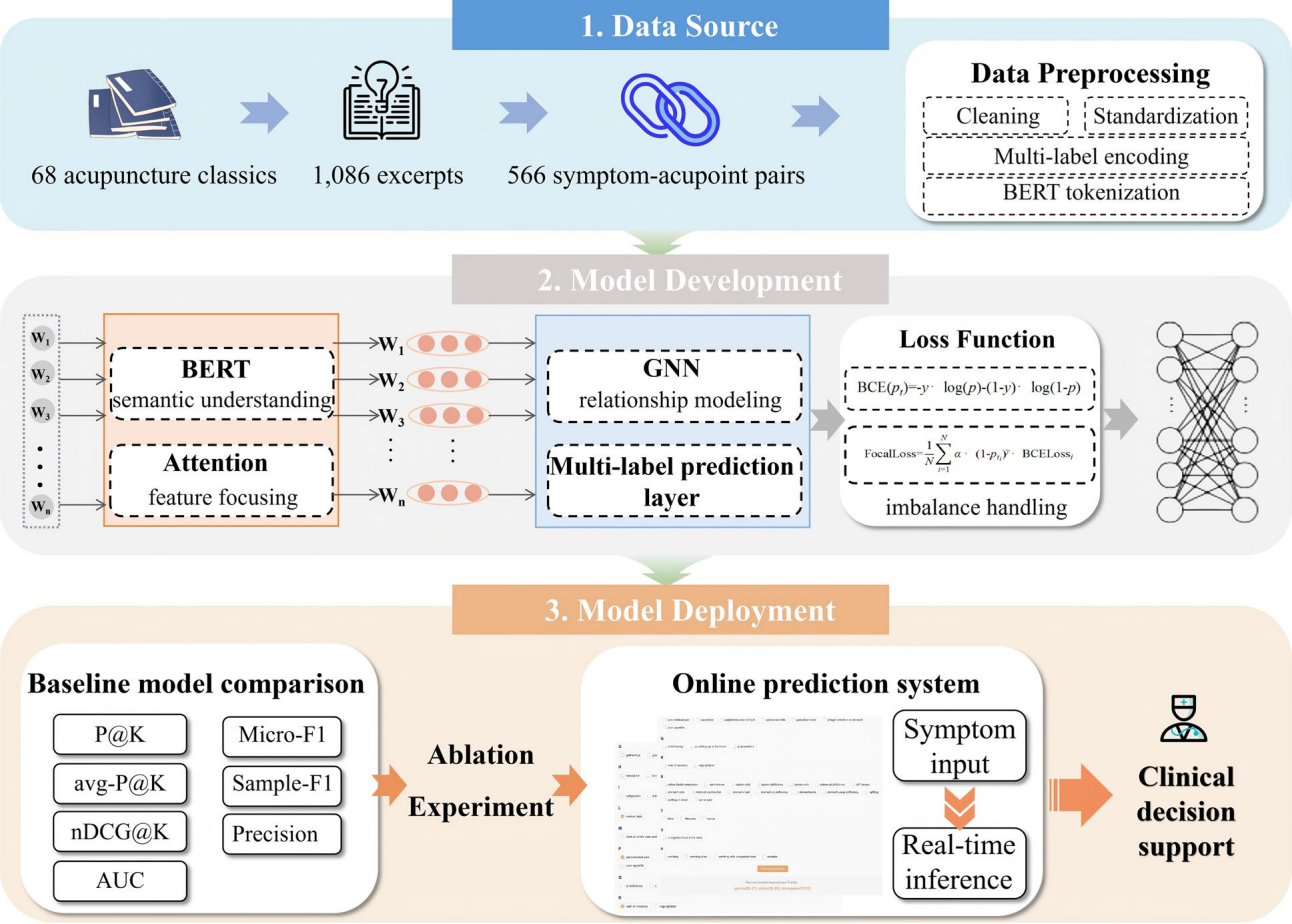
Flowchart of the manuscript structure

While these approaches demonstrated great potential, current data-driven approaches in acupuncture have not fully addressed the balance between complex acupoint compatibility and individualised treatment [19]. Many computational frameworks do not adequately incorporate core TCM theories, resulting in a disconnection between traditional principles and modern algorithms [20]. To address these challenges, we developed an MLTC framework that integrates heterogeneous graph structures and label co-occurrence information. Specifically, the framework employs BERT as a text encoder to extract semantic features, combined with an attention mechanism to emphasise key textual segments. Text nodes and learnable label nodes are jointly embedded to construct a text-label heterogeneous graph, where heterogeneous feature interaction is modelled using GNN [21]. In the decoding phase, a label fusion mechanism propagates information through graph convolutions based on the label co-occurrence probability matrix, iteratively refining the confidence of predictions. To enhance models’ robustness, a composite loss function is designed to mitigate class imbalance, and random token deletion noise is introduced during training [22]. To validate the clinical applicability of this approach, the trained PyTorch model was encapsulated via Flask and deployed as an online service [23], enabling real-time acupoint inference for user-input symptoms. The system integrates BERT-based text encoding, attention-weighted feature representation and GNN-enhanced relationship modelling while utilising Focal Loss to strengthen the prediction robustness for rare acupoint combinations. This engineering implementation demonstrates the model’s potential as an intelligent auxiliary tool for clinical practice, providing physicians with reference acupoint recommendations. The framework of the methodology of this paper is shown in Fig. 1.

## Materials and methods

**Fig. 2:**
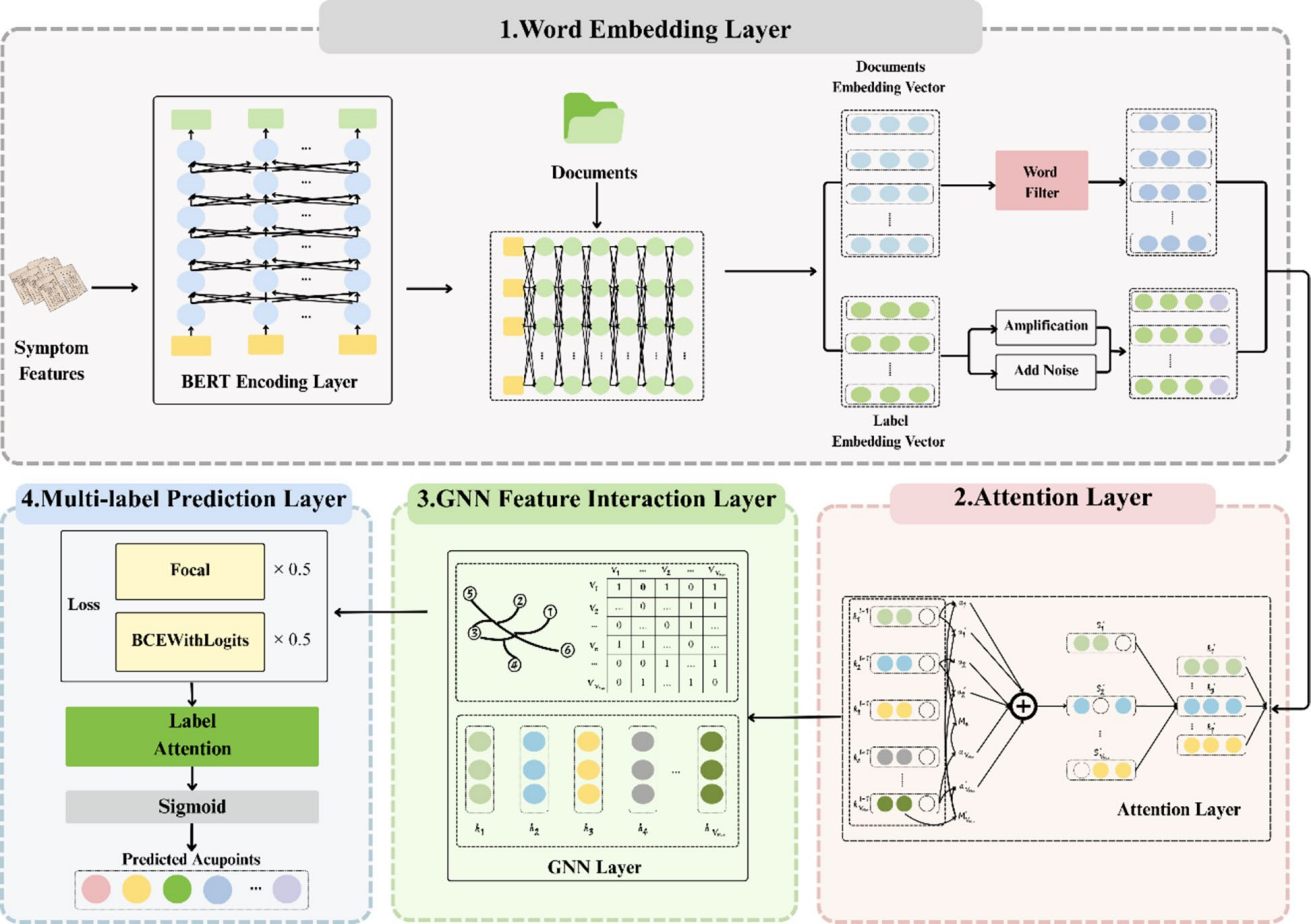
Schematic overview of the model

The multi-label classification model proposed in this paper consists of four main components: the word embedding layer, the attention layer, the GNN feature interaction layer and the multi-label prediction layer. The overall framework of the model is illustrated in Fig. 2.

### Data collection and standardisation

We compiled 1,086 excerpts from 68 classical acupuncture texts, including the *Miraculous Pivot* and *A–B Classic of Acupuncture and Moxibustion* [24], sourced from the Library of Chengdu University of TCM and digital archives such as IMEDBOOKS. Based on the Rome IV criteria [25] and TCM internal medicine guidelines [26], we identified eight core FGIDs symptom terms (e.g. abdominal pain, bloating and nausea). After the deduplication and removal of incomplete entries, 566 unique symptom-acupoint pairs were retained.

Symptoms’ terminology was standardised using the *Standardization of Common Symptom Terminology in Clinical Chinese Medicine* [27] and the *WHO International Standard Terminologies on Traditional Medicine* [28], consolidating synonymous expressions into a uniform body part and sensation format. Acupoint names were normalised according to the *Fundamentals of Acupuncture* [29]. The final dataset comprises 154 symptoms and 89 acupoints, with common acupoints including *Zusanli* (ST36), *Zhongwan* (CV12) and *Sanyinjiao* (SP6). Table 1 provides a snapshot of these excerpts, illustrating detailed acupuncture prescriptions for each symptom group as a representative example of the full dataset. The complete dataset of 566 entries is available upon request from the corresponding author.

**Table 1:** Snapshot of 566 excerpts and associated acupoints for symptoms

Source	Excerpts	Symptoms	Acupoints
*Acupuncture and Moxibustion Classic for Nourishing the Life*	Nausea, vomiting after eating, abnormal rising of qi	regurgitation, vomiting	*Rugen* (ST18), *Shuifen* (RN9), *Rangu* (KI2)
*Arcane Essentials from the Imperial Library*	Accumulations and gatherings in five viscera and six bowels, five viscera and six bowels distention and fullness, deficiency and no desire to eat	accumulations, gatherings, abdominal distention, abdominal fullness, inappetence	*Sanjiaoshu* (BL22)
*Classic of Divine Responses*	Indigestion, fullness in the chest and abdomen, sallow facial complexion is called spleen and stomach disease	inappetence, epigastric and abdominal distention, sallow facial complexion	*Zhongwan* (CV12)
*Divine Moxibustion Classic*	Distending pain of heart and spleen	heart pain, spleen pain	*Shangwan* (RN13), *Zhongwan* (RN12), *Pishu* (BL20), *Weishu* (BL21), *Shenshu* (BL23), *Yinbai* (SP1)
*Four Medical Tantras*	Stomach distention, hiccup, stomachache, belching, borborygmus, and diarrhea	gastric distention, hiccup, stomachache, belching, borborygmus, diarrhea	*Weishu* (BL21)
*Guide to the Acupuncture Classics*	Cold in spleen, stomachache	stomachache	*Gongsun* (SP4)
*Great Compendium of Acupuncture and Moxibustion*	Acupuncture at laogong treats heat pattern of heart pain and regurgitation; cold pattern selects shaoze to tonify	heart pain, regurgitation	*Laogong* (PC8), *Shaoze* (SI1)
*Taiyi Divine Needle*	Cold pain in chest and abdomen, palpitations due to fright, intermingled phlegm and food stagnation, masses in the upper abdomen, accumulations and gatherings, concretions, abdominal distension, bowel sounds, indigestion	chest pain, palpitation, phlegm-food stagnation, epigastric stuffiness, accumulations, abdominal distention, borborygmus, indigestion	*Shangwan* (RN13), *Zhongwan* (RN12), *Danzhong* (CV17), *Zusanli* (ST36)
*Six Odes on Acupuncture and Moxibustion*	Indigestion due to spleen deficiency	indigestion	*Pishu* (BL20), *Pangguangshu* (BL28)
*Complete Book of Channels and Collaterals*	Pathogenic qi in spleen leads to vexation and inappetence; inappetence is caused by stagnation of stomach qi	inappetence, epigastric and abdominal distention, sallow facial complexion	*Zhongwan* (CV12)
…			

### Data preprocessing

Based on the data related to acupuncture treatment for FGIDs, which includes symptom descriptions and corresponding acupoint information, we formulated the symptom descriptions as input text and the acupoint information as multi-label outputs. To preprocess the data, we inject noise into the original symptom-acupoint data using random token deletion with a noise factor of 0.1. Subsequently, we employed dynamic label encoding via MultiLabelBinarizer to construct multi-hot label vectors, mapping the original comma-separated acupoint labels into a binary vector space. Finally, the symptom texts are tokenised using the BERT tokenizer (BERT-base-uncased), and the subwords are padded to a uniform length of 128 tokens while generating the attention mask matrix, forming structured inputs.

### Input encoding layer

To capture the sequential, structural and semantic information of symptom texts, it is necessary to model irregular text data. We construct a graph structure that reflects the compatibility relationships between acupoints, encoding prior knowledge from TCM, such as classic acupoint pairs, meridian connectivity and the synergistic effects of acupoints. Each symptom in the text is treated as an edge, connecting the syntactic structures of symptoms in the acupoint prescription. The edge weights are adjusted based on the symptoms to enhance the relevant acupoint combinations. To further capture the semantic information, we employ the Dirichlet distribution to mine the topics of each symptom and use these topics as edges. Let $\left\{{y}_{1},{y}_{2}\dots \dots {y}_{N}\right\}$ (where N is the total number of acupoints) be the set of acupoint labels and define the initial adjacency matrix ${A}_{ij}$ (Eq. 1):

1 $$\begin{array}{*{20}c} {{\mathrm{A}}_{{{\mathrm{ij}}}} { = }\left\{ {\begin{array}{*{20}l} {1,} \hfill & {{\text{If }}\;{\mathrm{cupoints}}\;{\mathrm{y}}_{{\mathrm{i}}} \;{\mathrm{and}}\;{\mathrm{y}}_{{\mathrm{j}}} \;{\mathrm{satisfy}}\;{\mathrm{the}}\;{\mathrm{condition}}\;{\mathrm{of}}\;{\mathrm{classic}}\;{\mathrm{acupoint}}\;{\mathrm{pairing}}} \hfill \\ {} \hfill & {{\mathrm{or}}\;{\mathrm{anatomical}}\;{\mathrm{adjacency}}} \hfill \\ {0,} \hfill & {{\mathrm{Otherwise}}} \hfill \\ \end{array} } \right.} \\ \end{array}$$

### BERT semantic extraction

The BERT pretrained language model has made significant contributions to NLP. It leverages the Encoder layers of the Transformer model to extract features from the text sequences to be predicted. Numerous studies have demonstrated that utilising the BERT model to obtain initial representations of text words can significantly improve the accuracy of MLTC tasks. Therefore, we adopt the BERT model as the word-embedding extractor.

In addition, we use the BERT-base-uncased BERT tokenizer, BertTokenizer, to tokenize the symptom description texts and convert them into corresponding token IDs [30]. By employing bidirectional Transformer encoders, context-aware token embeddings are generated that better capture the semantics of the words in the symptoms. Special tokens [CLS] and [SEP] are added, and the corresponding attention mask is generated. The original text sequences are concatenated with the symptoms and input into the BERT model in the form of auxiliary sentence pairs. The input format is depicted in Eq. 2:

2 $${\mathrm{Input}} = [{\mathrm{CLS}}] + {\mathrm{S}} + [{\mathrm{SEP}}] + a_{i} + [{\mathrm{SEP}}]$$

where [CLS] is a special token in the BERT model that represents the beginning of the symptom, *S* is the tokenized symptom description sequence, [SEP] is a special token used to separate symptoms, and a_i_ is the tokenized name of the i-th candidate acupoint (i  ∈{1, 2, …, N} and N denotes the total number of acupoints).

### Attention layer

In the task of MLTC for TCM texts, the attention layer helps the model focus on key terms in the symptom descriptions (such as abdominal bloating and dull pain) by dynamically allocating weights and establishing their association with specific acupoints, such as *Zusanli* (ST36) and *Zhongwan* (CV12) [31]. Let the hidden state matrix *H* obtained after encoding the symptom description with BERT be $H=[{h}_{1},{h}_{2},\dots ,{h}_{T}]\in {R}^{T\times 768}$, where each token corresponds to a 768–dimensional vector and *T* is the sequence length.

A nonlinear transformation is performed through a learnable parameter matrix (Eq. 3):

3 $$e_{i} = \tanh (W_{a} h_{i} + b_{a} ) \cdot v_{a}$$

where ${W}_{a}\in {R}^{768\times T}$ is the weight matrix, ${v}_{a}$ is the projection vector and ${b}_{a}$ is the bias term, resulting in a score vector of $v=\{{e}_{1},{e}_{2},\dots ,{e}_{T}\}$. By simulating the clinical process of acupoint selection in acupuncture, nonlinear transformation extracts the diagnostic value of symptom terms.

Specifically, the attention weights are computed over the BERT hidden states $H \in { R}^{T\times 768}$, where *T* is the sequence length and 768 is the hidden state dimension.

4 $$\begin{array}{*{20}c} {u_{t} = \tanh (W_{a} h_{t} + b_{a} )} \\ \end{array}$$

5 $$\begin{array}{*{20}c} {\alpha_{t} = \frac{{\exp (v_{a}^{T} u_{t} )}}{{\mathop \sum \nolimits_{j = 1}^{T} \exp (v_{a}^{T} u_{j} )}}} \\ \end{array}$$

6 $$\begin{array}{*{20}c} {s = \mathop \sum \limits_{t = 1}^{T} \alpha_{t} h_{t} } \\ \end{array}$$

where ${h}_{t}\in {R}^{768}$ denotes the hidden representation of the t-th token in the input sequence.${W}_{a}\in {R}^{{d}_{att}\times 768}$ is the projection matrix, where ${d}_{att}=768$ is the attention hidden dimension, and ${b}_{a}\in {R}^{{d}_{att}}$ is the bias vector. The projected representation is denoted as producing ${u}_{t}\in {R}^{768}$. Moreover, ${v}_{a}\in {R}^{{d}_{att}}$ is the attention query vector, and ${v}_{a}^{T}{u}_{t}$ produces a scalar attention score. Next, ${\alpha}_{t}$ denotes the normalised attention weight assigned to the $t$-th token, and $s\in {R}^{768}$ represents the final attention-weighted semantic representation of the symptom.

The SoftMax function is applied to generate a probability distribution, normalising attention weights and aggregating key information through weighted summation to synthesise the context vector *c* (where $c\in {R}^{768}$) based on the symptoms.

7 $$\begin{array}{*{20}c} {c = \mathop \sum \limits_{i = 1}^{T} h_{i} \frac{{\exp (e_{i} )}}{{\mathop \sum \nolimits_{j = 1}^{T} \exp (e_{j} )}}} \\ \end{array}$$

### GNN feature interaction and fusion layer

In the knowledge system of TCM acupuncture, there are complex compatibility relationships and meridian connections between acupoints. By constructing an acupoint graph, this prior knowledge can be encoded into the model to enhance the rationality of feature representation [32]. The 768-dimensional global semantic vector was integrated with the acupoint graph structure, and feature transformation and dimensionality reduction were performed using GNN.

This paper defines a graph G = (V,E), where nodes refer to acupoint labels and edges are constructed based on the prior co-occurrence information of labels. If there is a synergistic relationship between the two labels, the corresponding nodes are connected by an edge. Node features are initialised as 256-dimensional random embedding vectors. Let A be the adjacency matrix of the acupoint relationship graph, and ${H}^{(0)}$ represent the initial node feature matrix. The global symptom semantics were mapped onto the acupoint graph space, and the node features were updated using GNN.

8 $$\begin{array}{*{20}c} {c_{proj} = W_{p} c + b_{p} (W_{p} \in R^{256 \times 768} ,\;b_{p} \in R^{256} )} \\ \end{array}$$

9 $$\begin{array}{*{20}c} {H^{(1)} = \sigma \left( {\hat{D}^{ - 1/2} \hat{A}\hat{D}^{ - 1/2} (H^{(0)} \oplus c_{proj} )W_{g} } \right)} \\ \end{array}$$

where $\widehat{A}$ denotes the adjacency matrix with added self-loops, $\widehat{D}$ is the degree matrix, $\oplus$ indicates the concatenation of cproj with the features of each acupoint label, ${W}_{g}$ is the learnable weight, and $\sigma$ is the ReLU activation function. The projected symptom vector ${c}_{proj}$ $\in {R}^{256}$ was first broadcast to all N acupoint nodes, resulting in an N $\times$ 256 symptom feature matrix. This matrix was then concatenated with the initial acupoint node feature matrix ${H}^{(0)}\in {R}^{N\times 256}$, yielding $X\in {R}^{N\times 512}$. Accordingly, the GNN weight matrix was defined as ${W}_{g}\in {R}^{512\times 256}$, producing the updated node representation ${H}^{(1)}\in {R}^{N\times 256}$.

### Multi-label prediction layer

The 256-dimensional acupoint features are mapped onto the space of *N* acupoint labels, supporting independent predictions for multiple labels, which aligns with the clinical prescription principle of “primary acupoints + auxiliary acupoints” in acupuncture [33]. If the output features from the GNN are denoted as ${H}^{(1)}$ and $\{{y}_{1},{y}_{2},\dots ,{y}_{N}\}$ represents the set of acupoint labels, a binary classification is performed for each acupoint node, yielding the unnormalized logit vectors $logit{s}_{i}$. The activation probability ${p}_{i}$ for each acupoint is independently calculated using the sigmoid function without enforcing the sum of probabilities at 1, allowing the simultaneous activation of multiple acupoints.

10 $$\begin{array}{*{20}c} {{\mathrm{logits}}_{i} = W_{fc}^{(i)} h_{i}^{(1)} + b_{fc}^{(i)} } \\ \end{array}$$

11 $$\begin{array}{*{20}c} {p_{i} = \sigma ({\mathrm{logits}}_{i} ) = \frac{1}{{1 + e^{{ - {\mathrm{logits}}_{i} }} }}} \\ \end{array}$$

where ${W}_{fc}^{(i)}$ and ${b}_{fc}^{(i)}$ are the independent parameters for the i-th acupoint.

### Dynamic loss function

To address the issue of a high imbalance between symptoms and acupoint labels, especially when dealing with long-tailed label distributions, we employ Focal Loss for loss function management [34]. Focal Loss mitigates the impact of easily classified samples on the loss by adding a focusing factor $BCE({p}_{t})$ to binary cross-entropy (BCE), allowing the model to concentrate more on hard-to-classify samples. It is particularly effective for class imbalance problems, balancing the loss contributions of positive and negative samples and optimising the model’s learning process by adjusting $\alpha$ and $\gamma$.

The BCE Loss function measures the difference between predicted probabilities and actual labels with the formula as follows:

12 $${\mathrm{BCE}}(p_{t} ) = - y \cdot \log (p) - (1 - y) \cdot \log (1 - p)$$

where *y* is the actual label, with a value of 0 or 1. Moreover, *p* is the model’s predicted probability output, calculated through the sigmoid activation function, representing the probability that the sample belongs to the positive class (1).

13 $$\begin{array}{*{20}c} {{\mathrm{FocalLoss}} = \frac{1}{N}\mathop \sum \limits_{i = 1}^{N} \alpha \cdot (1 - p_{{t_{i} }} )^{\gamma } \cdot {\mathrm{BCELoss}}_{i} } \\ \end{array}$$

where $\alpha$ is a weight coefficient that adjusts the imbalance between positive and negative samples. For instance, when there are fewer positive samples, $\alpha$>1 can be set to increase the weight of positive samples; $\gamma$ is the focusing factor that controls the loss weight of hard-to-classify and easily classified samples. A larger $\gamma$ will decrease the loss for easily classified samples, focusing on hard-to–classify ones; $(1-{p}_{t}{)}^{\gamma }$ is the modulation factor, where ${p}_{t}$ is the model’s predicted probability, indicating the confidence of the prediction for the sample, specifically:

14 $$p_{t} = \left\{ {\begin{array}{*{20}l} p \hfill & {y = 1} \hfill \\ {1 - p} \hfill & {y = 0} \hfill \\ \end{array} } \right.$$

The hyperparameters $\alpha$ and $\gamma$ in Focal Loss are used to balance class imbalance and easy/hard samples, respectively. In our study, these parameters were tuned through a grid search on the validation set. We searched over $\alpha \in${0.25, 0.50, 0.75, 1.00} and $\gamma \in${1.0, 1.5, 2.0, 2.5}. The model achieved the best evaluation performance in Micro F1 and Sample F1, when $\alpha$ = 1.0 and $\gamma$ = 2.0.

### Evaluation metrics

We adopt precision at K (P@K), average precision at K (avg-P@K), normalised discounted cumulative gain (nDCG@K), area under the ROC curve (AUC), micro-F1, sample-F1 and label-wise accuracy [35]. Baseline models include convolutional neural network–recurrent neural network (CNN–RNN), TextCNN, GNN-only and attention-only variants. To comprehensively evaluate the effectiveness of the proposed model, we compared GNN–BERT–Attention with several additional models, including general deep learning models, non-graph label fusion models, graph-structure ablation models and traditional TCM-related recommendation methods. These comparisons were designed to assess not only the overall predictive performance of the proposed framework but also the contribution of the graph topology, semantic representation, attention mechanism and domain-specific prior knowledge. In alphabetical order, the specific models and their intended functions are as follows:

- CNN-Attention [37]: uses convolutional networks to extract local textual features and applies attention to emphasise the symptom terms most relevant to acupoint prediction.
- CNNRNN [36]: combines convolutional layers for local symptom feature extraction with recurrent layers for sequential dependency modelling.
- Co-occurrence recommendation [46]: recommendation ranks acupoints according to their observed co-occurrence frequency with symptoms in the training data.
- Long short-term memory–BERT–Attention (LSTM–BERT–Attention) [42]: uses BERT and attention for symptom encoding and models label interactions through an LSTM-based non-graph sequence structure.
- Multi-layer perceptron-BERT-Attention (MLP-BERT-Attention) [41]: uses BERT and attention for symptom representation, but replaces graph-based label interaction with fully connected MLP label fusion.
- Random Graph GNN [43]: retains the GNN architecture but replaces the clinically informed acupoint graph with randomly generated edges to test the value of the real graph topology.
- RNN-Attention [38]: captures sequential symptom information using recurrent networks and employs attention to highlight key symptom representations.
- Rule-based reasoning [44]: recommends acupoints according to predefined symptom-acupoint mapping rules derived from clinical knowledge or classical literature.
- Term frequency-inverse document frequency-support vector machines (TF–IDF–SVM) [45]: represents symptom texts with TF–IDF features and uses a SVM for multi-label acupoint classification.
- TextCNN [39]: applies convolutional filters of different window sizes to extract n-gram features from symptom texts for multi-label classification.
- TextCNN-Attention [40]: enhances TextCNN by adding an attention mechanism to assign higher weights to clinically important symptom features.

## Results

### Experimental settings

The model was trained using Python 3.11.8. The key hyperparameters used for model training were configured as follows: The model was optimised using the Adam optimiser with an initial learning rate of 1 $\times {10}^{-4}$. The batch size was set to 16, and the model was trained for 300 epochs. The hidden dimension of the GNN node’s representations was set to 256. For text encoding, the maximum sequence length was set to 128, and BERT-base-uncased was adopted as the pre-trained BERT encoder to generate 768-dimensional contextual representations. To evaluate the model’s performance, the dataset was randomly divided into the train and test sets at a ratio of 8:2 using a fixed random seed of 42. This fixed partitioning strategy was adopted to ensure that all compared models were trained and evaluated under the same data distribution, thereby augmenting the reproducibility and fairness of the model comparison.

### Model performance comparison

**Fig. 3:**
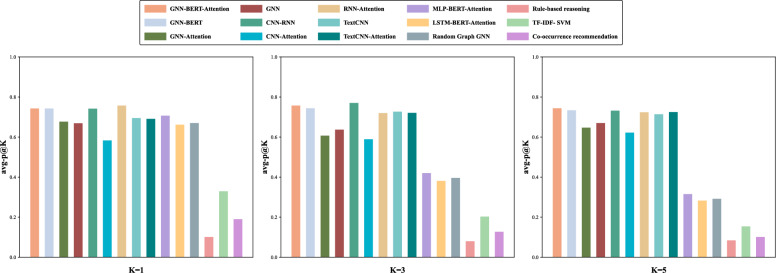
Comparison of avg_P@K in MLTC methods

As illustrated in Fig. 3, the comparison of avg–P@K (K = 1, 3, 5) across models indicates that the proposed GNN–BERT–Attention model consistently achieves superior performance, particularly in avg–P@3 and avg–P@5, which may demonstrate its strong ability to capture multiple relevant acupoints in multi–label prediction tasks. While most baseline models exhibit a decline in performance as K increases, reflecting the trade–off between precision and coverage, the proposed model maintains relatively stable and high values across all K levels. These observations imply that integrating a graph structure, semantic representation and attention mechanisms may be effective at ameliorating both the accuracy of core acupoint recommendations and the coverage of auxiliary acupoints, aligning well with the clinical practice of combined acupoint selection.

The slightly higher avg–P@3 than avg–P@5 reflects the inherent trade–off between recommendation coverage and precision as K increases, rather than directly indicating a problem with the ranking logic in Fig. 3. In clinical acupuncture practice, prescriptions usually comprise core and auxiliary acupoints. The top–3 predictions may mainly correspond to high–confidence core acupoints, whereas the top–5 predictions include additional auxiliary candidates. These additional candidates may be clinically plausible but may not always appear in the annotated label set, which can lead to a lower avg–P@5.

**Fig. 4:**
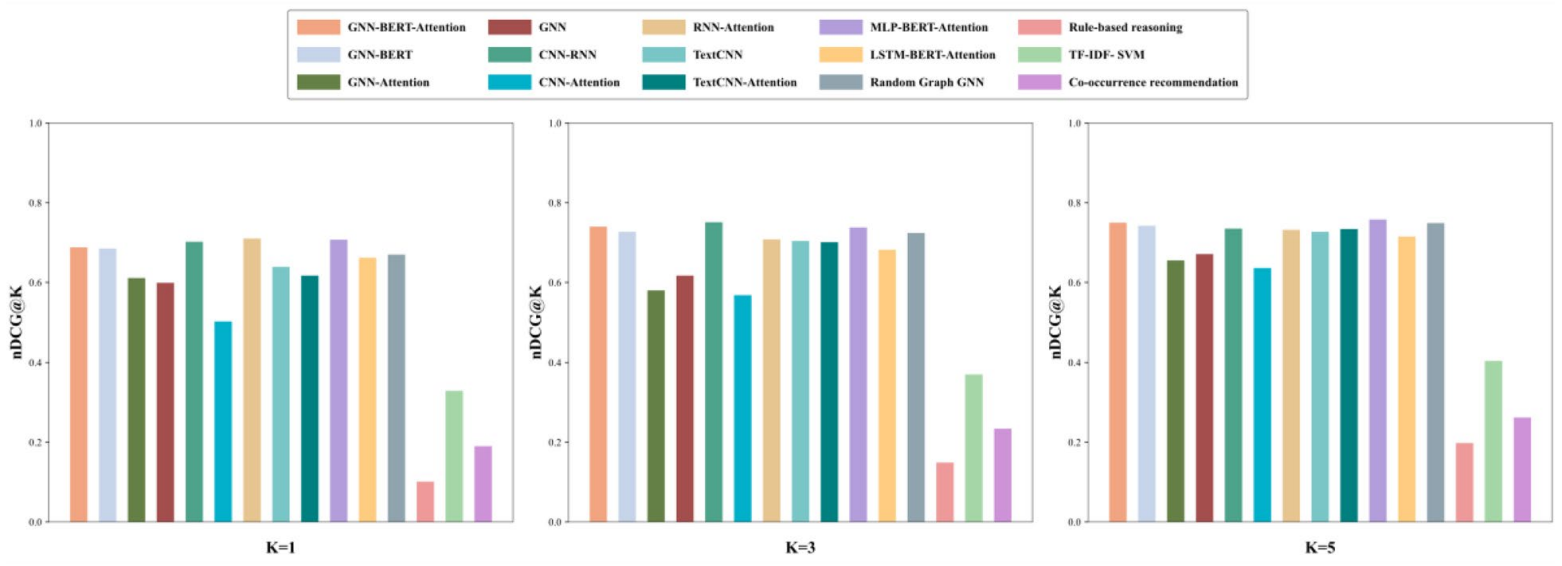
Comparison of nDCG@1, 3, 5 in MLTC methods

As shown in Fig. 4, the proposed GNN–BERT–Attention model achieved consistently high nDCG@K values across K = 1, K = 3 and K = 5, indicating its potential ranking ability in symptom–to–acupoint multi–label prediction. In particular, the model maintained competitive performance at nDCG@1 and revealed stable results as K increased, suggesting that it could identify relevant acupoints while preserving the quality of multi–acupoint recommendations. Compared with traditional machine–learning methods, such as TF–IDF–SVM, rule–based reasoning and co–occurrence recommendation, deep learning–based models tended to demonstrate better ranking performance. Among these, models incorporating BERT, graph structures or attention mechanisms performed more favourably, which appears to support the contribution of semantic representation learning and label–relation modelling. These results seem to suggest that integrating symptom semantics, acupoint graph associations and attention–based feature weighting may help refine the relevance ranking of predicted acupoints, which is important for generating recommendation lists in multi–label acupoint selection.

**Fig. 5:**
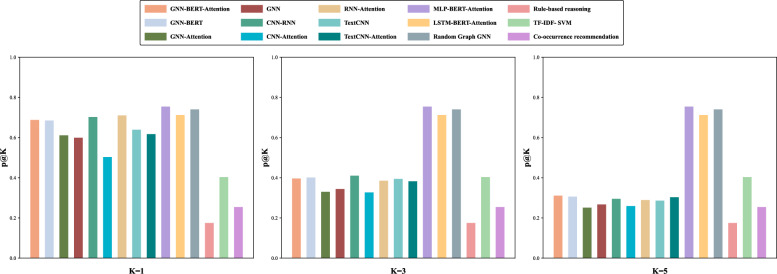
Comparison of P @ 1, 3, 5 in MLTC methods

As shown in Fig. 5, the P@K values decreased as K increased from 1 to 5 for most learning–based models, which seems to indicate that the prediction precision was highest when only the top–ranked label was considered and gradually declined when more candidate labels were included. For the proposed GNN–BERT–Attention model, P@1 reached 0.688, while P@3 and P@5 decreased to 0.396 and 0.311, respectively. A similar trend was observed for GNN–BERT, with values declining from 0.685 at K = 1 to 0.401 at K = 3 and 0.306 at K = 5. CNN–RNN showed the highest P@1 among the conventional neural models at 0.702. However, its performance also decreased to 0.410 and 0.295 at K = 3 and K = 5. In contrast, MLP–BERT–Attention, LSTM–BERT–Attention and Random Graph GNN maintained relatively low values at K = 5, with MLP–BERT–Attention reaching 0.302, LSTM–BERT–Attention 0.299 and Random Graph GNN 0.292. Traditional methods displayed lower overall performance, with rule–based reasoning remaining at 0.114 across K = 1 and co–occurrence recommendation at 0.215, whereas TF–IDF–SVM remained moderate at 0.291. Overall, these results find that increasing K generally reduced precision for most models, potentially reflecting the greater difficulty of maintaining accurate recommendations when more labels were retrieved.

For a detailed comparison of the performance metrics, please refer to Table 2, which presents avg–P@K, P@K and nDCG@K for K = 1, 3, 5 of the MLTC and baseline methods on the acupuncture dataset. GNN–BERT–Attention remained relatively stable, with avg–P@K values of 0.743, 0.757 and 0.744 at K = 1, K = 3 and K = 5. For P@K, most neural network models, excluding rule–based reasoning, showed a decreasing trend from K = 1 to K = 5. For nDCG@K, most models displayed an increasing trend as K increased. GNN–BERT–Attention increased from 0.688 at K = 1 to 0.740 at K = 3. Traditional methods, including rule–based reasoning, TF–IDF–SVM and co–occurrence recommendation, remained lower across all three metrics, although their nDCG@K values increased slightly with a larger K. Overall, the results indicate that deep learning–based models generally achieve higher and more stable performance than traditional methods across different K settings.

**Table 2:** Avg–P@K, P@K, nDCG@K (K = 1, 3, 5) of MLTC and baseline methods on acupuncture dataset

Model	avg–P@K			P@K			nDCG@K		
	K = 1	K = 3	K = 5	K = 1	K = 3	K = 5	K = 1	K = 3	K = 5
GNN–BERT–Attention	0.743	0.757	0.744	0.688	0.396	0.311	0.688	0.740	0.750
GNN–BERT	0.743	0.744	0.734	0.685	0.401	0.306	0.685	0.727	0.742
GNN–Attention	0.677	0.607	0.647	0.611	0.330	0.251	0.611	0.580	0.655
GNN	0.669	0.637	0.670	0.599	0.344	0.267	0.599	0.617	0.671
CNN–RNN	0.742	0.770	0.732	0.702	0.410	0.295	0.702	0.751	0.735
CNN–Attention	0.583	0.589	0.622	0.503	0.327	0.259	0.503	0.569	0.636
RNN–Attention	0.757	0.720	0.724	0.710	0.385	0.289	0.710	0.708	0.732
TextCNN	0.695	0.727	0.714	0.639	0.394	0.286	0.639	0.704	0.727
TextCNN–Attention	0.691	0.721	0.725	0.617	0.382	0.303	0.617	0.701	0.734
MLP–BERT–Attention	0.679	0.734	0.722	0.667	0.384	0.302	0.667	0.710	0.728
LSTM–BERT–Attention	0.653	0.727	0.714	0.653	0.380	0.299	0.653	0.701	0.717
Random Graph GNN	0.653	0.712	0.699	0.647	0.372	0.292	0.647	0.696	0.705
Rule–based reasoning	0.114	0.251	0.298	0.114	0.139	0.122	0.114	0.199	0.239
TF–IDF– SVM	0.291	0.379	0.372	0.291	0.198	0.155	0.291	0.364	0.375
Co–occurrence recommendation	0.215	0.341	0.335	0.215	0.178	0.140	0.215	0.308	0.338

### ROC curve

**Fig. 6:**
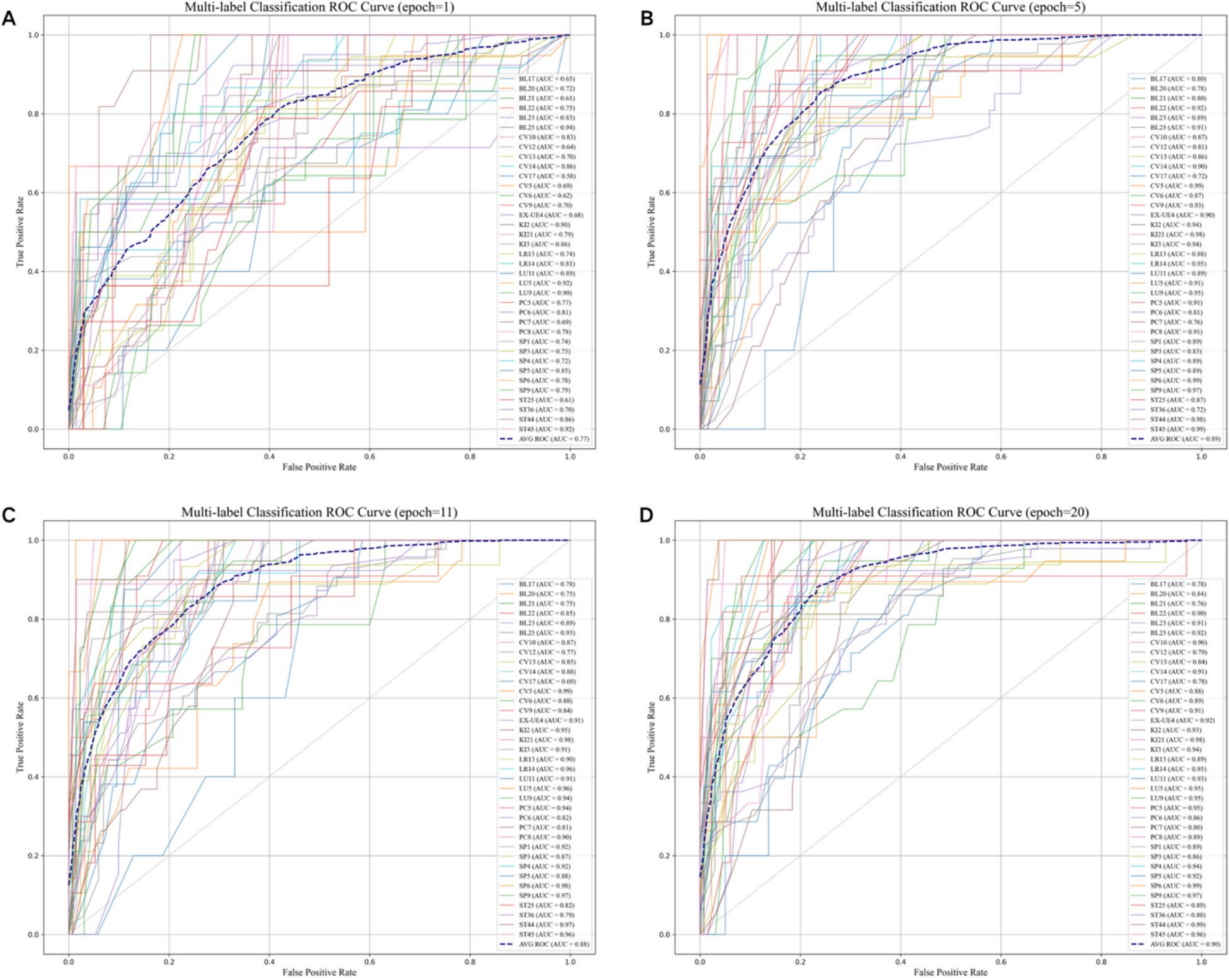
Multi–label Classification of ROC Curve. **A** epoch $=1$. **B** epoch $=5$. **C** epoch $=11$. **D** epoch $=20$

The receiver operating characteristic (ROC) curve and its corresponding area under the curve (AUC) values were used to assess the model’s ability to classify each acupoint in multi–label classification tasks from symptoms to acupoint mapping. As depicted in Fig. 6, the ROC curves of the acupoint labels suggest a progressive improvement in the performance of the GNN–BERT–Attention model as training proceeded from epoch 1 to epochs 5, 11 and 20. Specifically, the overall ROC profile gradually shifted towards the upper–left corner, tending to indicate enhanced discriminative ability across labels, and the mean AUC increased steadily from approximately 0.77 at epoch 1 to a higher level at epoch 5, followed by a further increase at epoch 11, eventually reaching about 0.90 at epoch 20. This continuous rise in average AUC suggests that the model may be achieving increasingly better classification performance during training and that its predictive capability appeared to become progressively more stable and reliable with additional epochs.

### Model interpretability

**Fig. 7:**
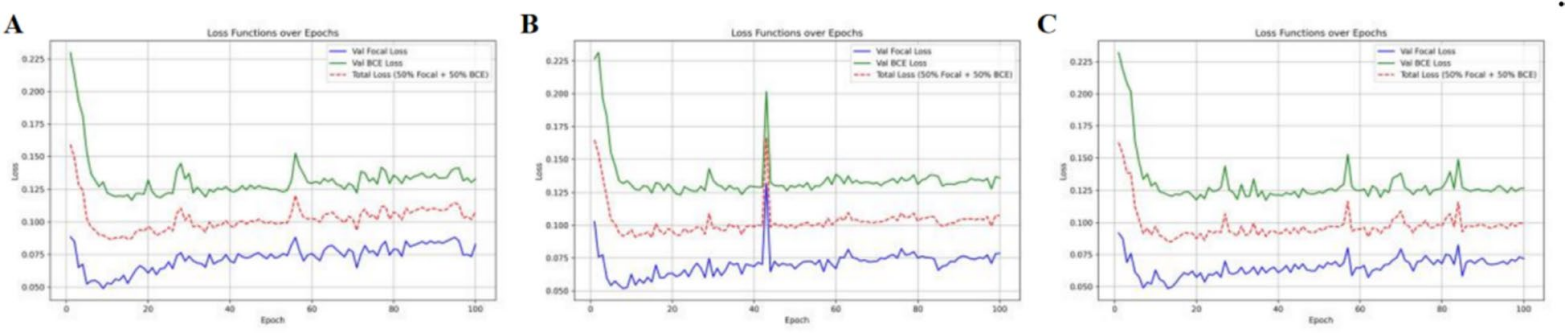
Rate of change in loss function. **A** $k=1$. **B** $k=2$. **C** $k=3$

Figure 7 illustrates the combined loss function of Focal Loss and BCE Loss during the training process. The stability and low values of the total loss indicate that this combination of loss functions is effective, guiding the model’s optimisation and enhancing performance. The clear trend of the loss function’s change suggests reliable experimental results. The GNN–BERT–Attention model gradually converges after approximately 10 epochs, with the total loss becoming stable. This indicates that the model can converge quickly during training and that the change in loss on the validation set is minimal, demonstrating good generalisation ability. Initially, both Focal Loss and BCE Loss decrease significantly, indicating that both effectively guide the model’s optimisation in the early stages of training; later, the stability of the total loss indicates the effectiveness of the combined loss function. Combined loss may incorporate cross–entropy loss and label correlation loss to alleviate the sparsity of label co–occurrence, such as *Zusanli* (ST36) and *Sanyinjiao* (SP6), which often appear together.

### Classification accuracy of different labels

The micro F1 score contributes equally to all labels, making it suitable for evaluation in the presence of sample imbalance [47]. It aggregates the prediction results across all labels, focusing on overall performance rather than calculating separately for each label. The sample F1 score calculates the F1 score independently for each sample (instance) and then takes the average of all sample F1 scores. It focuses on the prediction performance of each sample, considering the accuracy of each label within the sample. Sample F1 measures the model’s performance by averaging the F1 scores of each sample. This is particularly applicable to multi–label classification problems, especially when there is a significant variation in the number of labels across samples, as Sample F1 pays more attention to how the model handles each individual sample.

15 $$\begin{array}{*{20}c} {{\text{Micro - F}}1 = \frac{{2 \times {\mathrm{micro}}\_{\mathrm{precision}} \times {\mathrm{micro}}\_{\mathrm{recall}}}}{{{\mathrm{micro}}\_{\mathrm{precision}} + {\mathrm{micro}}\_{\mathrm{recall}}}}} \\ \end{array}$$

where micro_precision and micro_recall are calculated globally.

16 $$\begin{array}{*{20}c} {{\text{Sample - F}}1 = \frac{1}{N}\mathop \sum \limits_{i = 1}^{N} F1_{i} } \\ \end{array}$$

where $N$ represents the number of samples and $F{1}_{i}$ denotes the F1 score of the $i$–th sample.

The GNN–BERT–Attention model demonstrates optimal performance in the multi–label classification task of acupoint identification, depicting a relatively high proportion of correctly predicted positive acupoints/symptoms (as shown in Table 3). The proposed GNN–BERT–Attention model achieved the highest overall values across the evaluation metrics, with a label average accuracy of 0.960, micro F1 of 0.603, sample F1 of 0.559 and precision of 0.799. GNN–BERT revealed slightly lower but comparable results, with values of 0.955, 0.581, 0.557 and 0.753, respectively. Among the baseline neural models, CNN–RNN also obtained a high label average accuracy of 0.959; however, its micro F1 and sample F1 decreased markedly to 0.291 and 0.243. RNN–Attention presented relatively high micro F1 and sample F1 values of 0.583 and 0.559, although its precision was lower at 0.692. TextCNN and TextCNN–Attention achieved similar label average accuracy values of 0.953. However, their Micro F1 and Sample F1 values remained lower than those of GNN–BERT–Attention. In contrast, LSTM–BERT–Attention, MLP–BERT–Attention and Random Graph GNN showed much lower label average accuracy values, ranging from 0.062 to 0.074, although their micro F1 values remained approximately 0.454 to 0.477. Traditional methods performed relatively poorly, with rule–based reasoning, TF–IDF–SVM and co–occurrence recommendation exhibiting lower values across all metrics, particularly in Micro F1 and Sample F1. Overall, these results indicate that the GNN–BERT–Attention model tended to maintain the most balanced and comparatively higher performance among all the compared methods.

**Table 3:** Macro–averaged results for accuracy, micro–F1, sample–F1 and precision

Models	Label Average Accuracy	Micro F1	Sample F1	Precision
GNN–BERT–Attention	0.960	0.603	0.559	0.799
GNN–BERT	0.955	0.581	0.557	0.753
GNN–Attention	0.937	0.186	0.134	0.673
GNN	0.938	0.195	0.159	0.726
CNN–RNN	0.959	0.291	0.243	0.712
CNN–Attention	0.940	0.221	0.162	0.653
RNN–Attention	0.942	0.583	0.559	0.692
TextCNN	0.953	0.439	0.439	0.799
TextCNN–Attention	0.953	0.479	0.438	0.738
MLP–BERT–Attention	0.062	0.464	0.488	0.487
LSTM–BERT–Attention	0.062	0.454	0.486	0.491
Random Graph GNN	0.074	0.477	0.506	0.503
Rule–based reasoning	0.050	0.152	0.147	0.158
TF–IDF– SVM	0.050	0.256	0.263	0.266
Co–occurrence recommendation	0.050	0.238	0.229	0.247

This GNN–BERT–Attention model is attributed to the introduction of a hierarchical attention mechanism that correlates key symptoms with crucial acupoints, distinguishing between the primary and secondary relationships between symptoms and acupoints. However, its recall rate remains a limitation in practical applications. This may also relate to the combination of acupoints in acupuncture prescriptions. In TCM theory, the selection of acupoints is not only dependent on core symptoms but is also influenced by the overall syndrome differentiation and treatment principles. When combining acupoints, multiple acupoints are typically chosen based on the meridian pathways, the functions of the viscera and the interrelationships between symptoms for a synergistic therapeutic effect. This complex principle of acupoint combinations may cause the model to underestimate the importance of certain acupoints under specific symptoms, leading to a lower recall rate.

### Ablation experiment

To further verify whether the improvement was derived from the graph topology rather than merely from label embedding fusion, we designed a non–graph ablation variant. In this variant, the GNN–based feature interaction layer was replaced with a fully connected MLP and LSTM. As shown in Table 4, the ablation results demonstrate that incorporating the GNN module may significantly enhances the model’s overall performance. The GNN–BERT–Attention model achieves the highest label average accuracy of 0.960 and precision of 0.799, while maintaining a strong micro F1 of 0.603 and sample F1 of 0.559, indicating robust performance across both label–level and instance–level evaluations. In contrast, replacing the GNN component with MLP or LSTM leads to consistent degradation across all metrics, with labels’ average accuracy dropping sharply to 0.062, alongside noticeable declines in micro F1, sample F1 and precision. These results suggest that the GNN module plays a critical role in modelling structural dependencies, which may substantially improve the effectiveness of the classification task.

**Table 4:** Ablation study

Models	Label Average Accuracy	Micro F1	Sample F1	Precision
GNN–BERT–Attention	0.960	0.603	0.559	0.799
MLP–BERT–Attention	0.062	0.464	0.488	0.487
LSTM–BERT–Attention	0.062	0.454	0.486	0.491
GNN–BERT–Attention (BCE Loss)	0.412	0.464	0.548	0.688
GNN–BERT–Attention (No Augmentation)	0.050	0.292	0.278	0.323

Meanwhile, the ablation results demonstrated clear performance changes when key components were removed from the GNN–BERT–Attention model for acupoint compatibility recommendations. When the loss function was replaced with BCE Loss, the labels’ average accuracy decreased markedly to 0.412, while Micro F1, Sample F1 and precision dropped to 0.464, 0.548 and 0.688, respectively. A further decline was observed after removing data augmentation, with labels’ average accuracy, Micro F1, Sample F1 and precision decreasing to 0.050, 0.292, 0.278 and 0.323, respectively. These results appear to indicate that both the optimised loss function and data augmentation contributed to the model’s performance and their removal corresponded to a substantial reduction in the accuracy and stability of acupoint compatibility prediction.

These findings indicate that removing each key component can lead to different degrees of performance degradation. Replacing the GNN with an MLP or LSTM reduces the model’s ability to capture structured correlations among acupoints, indicating the contribution of graph topology. Both the optimised loss function and data augmentation likely contributed to the model’s performance, and their removal was associated with a substantial reduction in the accuracy and stability of acupoint compatibility prediction. In acupoint recommendation, these findings indicate that graph topology, imbalance–aware loss optimisation and data augmentation jointly may help the model capture clinically meaningful acupoint compatibility patterns, boost the recognition of relevant acupoint combinations from symptom descriptions and provide more reliable candidate recommendations for decision support.

### Kullback–Leibler divergence analysis

To assess the relationship between the predicted labels and the actual labels in both the training and test sets, we select Kullback–Leibler Divergence (KL–Divergence) [48], entropy and cross–entropy to calculate the distance between the predicted distribution ($\widehat{p}$) and the true distribution on the baseline dataset. The cross-entropy represents a label-summed value averaged over samples, meaning that the loss was summed across all labels for each sample and then divided by the number of samples rather than being further averaged by the number of labels. A smaller KL divergence indicates that the model’s predicted probability distribution is closer to the true distribution, suggesting better model calibration.

17 $$\begin{array}{*{20}c} {D_{KL} (Y\parallel \hat{Y}) = \mathop \sum \limits_{n = 1}^{N} \left[ {y_{n} \log \frac{{y_{n} }}{{\hat{y}_{n} }} + (1 - y_{n} )\log \frac{{1 - y_{n} }}{{1 - \hat{y}_{n} }}} \right]} \\ \end{array}$$

18 $$\begin{array}{*{20}c} {H(Y) = - \mathop \sum \limits_{n = 1}^{N} \left[ {y_{n} \log y_{n} + (1 - y_{n} )\log (1 - y_{n} )} \right]} \\ \end{array}$$

19 $$\begin{array}{*{20}c} {H(Y,\hat{Y}) = - \mathop \sum \limits_{n = 1}^{N} \left[ {y_{n} \log \hat{y}_{n} + (1 - y_{n} )\log (1 - \hat{y}_{n} )} \right]} \\ \end{array}$$

where ${y}_{n}$ represents the true value of the $n$-th label. Moreover, ${\widehat{y}}_{n}$ is the predicted probability of the *n*-th label, and $N$ denotes the total number of labels.

As shown in Table 5, the GNN-BERT-Attention model achieves relatively lower cross-entropy and KL divergence values than most baseline models, suggesting that its predicted label distribution is closer to the empirical true label distribution in symptom-to-acupoint multi-label classification. Specifically, the model obtains a test cross-entropy of 3.543 and a test KL divergence of 0.076, which may indicate better distributional consistency and prediction calibration. The relatively low entropy value also suggests that the model produces more stable prediction distributions. These results may be related to the integration of graph–based acupoint interaction modelling, BERT–based symptom semantic representation and attention–based key symptom extraction.

**Table 5:** Distribution–level comparison based on cross–entropy, entropy and KL divergence

Models	Cross-Entropy		Entropy		KL Divergence	
	Train	Test	Train	Test	Train	Test
GNN-BERT-Attention	4.304	3.543	0.099	0.097	0.128	0.076
GNN-BERT	4.764	4.636	0.268	0.285	0.163	0.147
GNN-Attention	6.147	6.258	0.481	0.478	0.278	0.126
GNN	6.872	6.103	0.483	0.480	0.283	0.267
CNN-RNN	4.723	4.491	0.262	0.274	0.151	0.140
CNN-Attention	6.412	5.974	0.402	0.419	0.239	0.248
RNN-Attention	4.680	4.454	0.244	0.257	0.154	0.142
TextCNN	4.843	4.980	0.319	0.319	0.173	0.169
TextCNN-Attention	5.263	4.671	0.301	0.288	0.174	0.154

### Model deployment

The deployment environment for the model is a server that supports Python and PyTorch, with relevant packages such as Flask, Torch, Transformers and scikit–learn installed. The application was developed based on the Flask framework, with an app.py script initiated in the terminal. The trained model was then loaded along with its weights. GET and POST requests are handled within the Flask application. When users submit symptoms via a web form, the system pre-processes these symptoms and inputs them into the model for prediction. The results are subsequently rendered through a template and returned to the user. The system can be accessed at http://47.109.102.62:12345/.

**Fig. 8:**
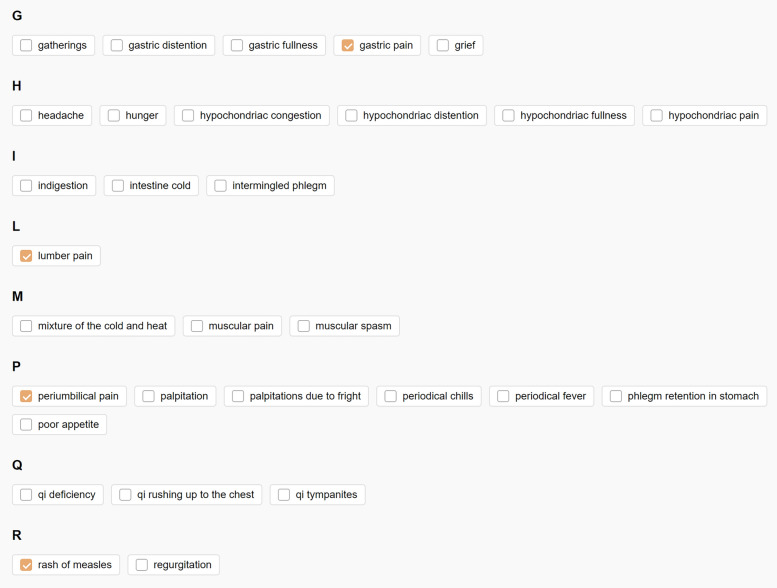
Symptom selection interface

**Fig. 9:**
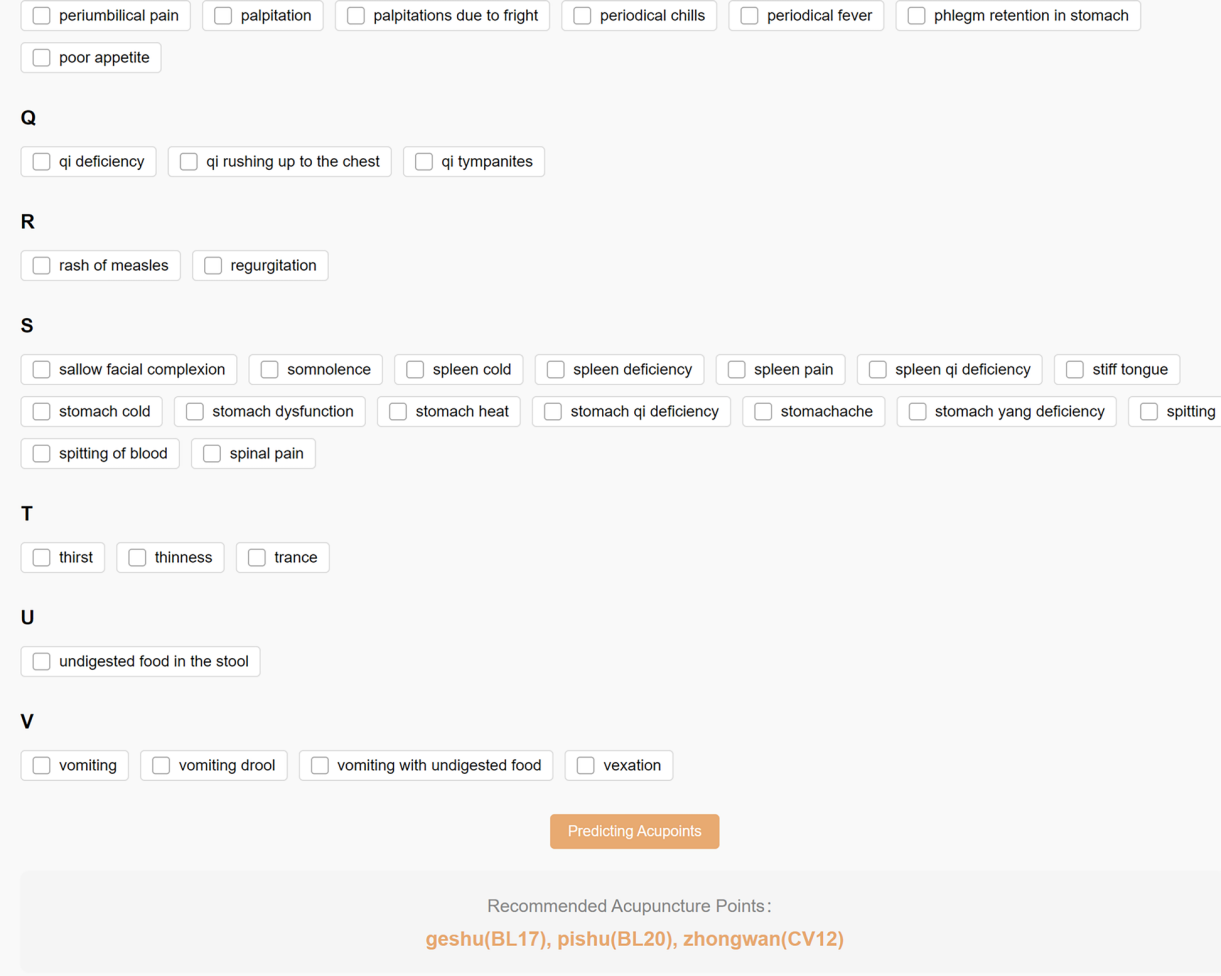
Acupoint prediction results interface

This system is a deep learning–based TCM symptom–acupoint prediction system designed to assist users in selecting related acupoints by inputting symptoms. It utilises a combination of BERT models, GNN and attention mechanisms for symptom classification. The main components include the Flask web framework, the PyTorch deep learning framework and a pre–trained version of the model. Users can select symptoms through the web interface, and the system predicts and returns relevant acupoint information. For instance, after selecting symptoms such as gastric pain, lumbar pain, peri lumbar pain and rash of measles (Fig. 8) and clicking the prediction button, the results are returned as portrayed by Fig. 9.

Upon symptom inputting, the system uses the BERT model to encode text, transforming it into embedding representations. Subsequently, a custom attention layer extracts key information, enhancing the model’s ability to capture complex relationships between symptoms. This information is then passed to a GNN layer, which extracts deeper features and is finally classified through a fully connected layer. To heighten the model’s robustness, the system also integrates Focal Loss, an optimised loss function for class imbalance issues, effectively addressing the scarcity of many symptom labels.

## Discussion

This study proposes the MLTC framework based on GNN integrated with a BERT–attention mechanism for predicting acupoint compatibility in FGIDs. By combining semantic understanding, relational reasoning and prior clinical knowledge from TCM, the model achieves superior performance across multiple evaluation metrics and provides real–time, interpretable acupoint recommendations through a deployed web system. Below, we discuss this work along three key dimensions: data standardisation and structured knowledge modelling, heterogeneous feature interaction and interpretability mechanisms, and the clinical applicability of the model and the significance of the system’s deployment.

The symptom–acupoint heterogeneous graph constructed in this study provides an important foundation for the standardisation and structured modelling of acupuncture knowledge. By integrating symptom–acupoint correspondences from 68 classical texts and unifying terminology according to the *Standardization of Common Symptom Terminology in Clinical Chinese Medicine* and the WHO’s *International Standard Terminologies*, this dataset enables a transition from unstructured text to a structured knowledge network. In particular, by constructing a graph with symptoms as edges and acupoints as nodes [49] and encoding classic pairing principles (e.g. the back–shu and front–mu association, distant–local point pairing) into the adjacency matrix, the model not only enhances the rationality of feature representation but also provides explicit prior constraints for subsequent GNN inference. Compared to previous methods that rely solely on textual co‑occurrence or statistical association, this structured modelling approach that incorporates TCM theory aligns more closely with the holistic and synergistic logic of “main points + auxiliary points” in acupuncture prescriptions, offering a computable foundation for uncovering implicit compatibility patterns from data.

At the level of feature extraction and interaction, this study achieves the multi‑level integration of textual semantics, local attention and global topological relations through a cascaded BERT-attention-GNN architecture [50]. The BERT encoder extracts context‑aware semantic features from symptom descriptions; the attention mechanism dynamically weights key symptom terms (e.g. abdominal distension and dull pain), simulating the thought process of TCM syndrome‑based acupoint selection. The GNN performs feature propagation and aggregation on the acupoint relation graph, capturing synergistic, meridian‑related and functional associations among acupoints [51]. This design not only improves model performance in terms of precision and coverage but also provides a certain degree of interpretability through attention weights and graph node updates [52]. For instance, the model assigns high attention weights to acupoints such as *Zusanli* (ST36) and *Zhongwan* (CV12) [53] when predicting gastric pain, which is consistent with common clinical practice. Notably, there remains room for improvement in recall, which may relate to the fact that complex TCM principles such as syndrome differentiation and treatment, meridian circulation are not yet fully captured by the graph structure. Future work could consider incorporating additional TCM diagnostic dimensions, such as syndrome elements and constitutional types, to further enrich the semantic layers of the graph.

This study also demonstrates the clinical potential of the model through an online demonstration system (http://47.109.102.62:12345/). The system allows users to input symptom descriptions and returns real‑time acupoint recommendations, showing good interactivity and response speed. This provides TCM practitioners with an auxiliary decision‑making tool, which is particularly useful in scenarios such as initial prescription drafting, teaching and training, or telemedicine. Moreover, to address class imbalance, the Focal Loss and random token deletion strategies adopted in this study effectively mitigate the impact of long‑tail labels on model performance, maintaining relatively stable prediction capability, even for low‑frequency acupoint combinations. The results show that the model demonstrated satisfactory performance on high‑frequency acupoints, such as *Zusanli* (ST36) and *Zhongwan* (CV12), indicating its ability to effectively learn common treatment patterns, which align closely with clinical practice.

The methodological contribution of this work does not lie in proposing an entirely new deep-learning algorithm. Instead, the main contribution lies in the systematic integration and domain adaptation of these techniques for the specific task of predicting acupoint compatibility. By formulating symptom-to-acupoint recommendations as an MLTC problem and incorporating acupoint compatibility knowledge through a graph-based label interaction module, this study provides a practical computational framework for modelling symptom-acupoint associations in acupuncture prescriptions. In the future, we plan to extend this methodology to other diseases, exploring optimisation strategies for acupuncture prescriptions in various clinical contexts.

Indeed, recent advances in deep nonlinear factorisation [54] and frequency-domain attention [55] offer valuable insights for extending this framework. These methods have demonstrated effectiveness at extracting robust latent features [56], enhancing key representations and handling noisy [57], high-dimensional [58] and incomplete biomedical data [59]. Although our task differs from fMRI analysis, symptom-acupoint data share similar challenges, including sparsity, non-linearity and complex label interactions. Future work could integrate tensor-based nonlinear factorisation with frequency--domain attention to better capture high-order compatibility patterns and recover missing associations. Additionally, stability analysis frameworks from these studies may help evaluate the robustness and reliability of TCM acupoint recommendation models.

## Limitations

This study integrates symptoms’ semantic representation, attention-based key symptom extraction, graph-based acupoint interaction modelling and multi-label learning to provide a domain–adapted deep learning framework for acupoint compatibility prediction. Despite the progress made in several aspects, this study still has certain limitations. The data scale was relatively modest, comprising 566 symptom-acupoint pairs, which constrains the model's generalisation ability and coverage breadth. Moreover, although an internal train/test split was used, external or multi-centre cross-validation was not conducted, mainly because of the limited sample size and sparse multi-label distribution, and repeated k-fold splitting may have resulted in unstable label distributions across folds. Therefore, the model should currently be regarded as an auxiliary decision-support tool rather than as a substitute for clinical judgment. To address these issues, future studies should expand the dataset with larger-scale modern medical records and electronic health records, include external validation cohorts, improve the modelling of rare acupoints, and assess the clinical applicability of the recommended acupoint combinations in real-world practice.

Another limitation lies in the graph structure, which is primarily built on documented acupoint pairing relationships and does not yet fully incorporate dynamic TCM theoretical relations, such as meridian circulation and the five-phase interactions. In addition, while interpretability is partially achieved through the attention mechanism and graph structure, deeper theoretical explanations for why a particular acupoint is selected are still lacking. Subsequent studies could introduce dynamic graphs or knowledge graphs to supplement the graph structure, and incorporate causal reasoning modelling based on TCM syndrome mechanisms [60] to enhance theoretical interpretability.

## Conclusion

We propose a GNN-BERT-attention framework for multi-label acupoint prediction in FGIDs acupuncture. By combining semantic understanding, relational reasoning and prior clinical knowledge, the model achieves superior performance and provides interpretable, real-time recommendations through a deployed web system. This work advances the data-driven modernisation of acupuncture prescriptions, supports clinical decision making and lays the groundwork for personalised, evidence-based TCM interventions.

## Data Availability

The datasets used and/or analysed during the current study are available from the corresponding author on reasonable request.

## Abbreviations

FGIDs: Functional gastrointestinal disorders
TCM: Traditional Chinese medicine
MLTC: Multi-label text classification
NLP: Natural language processing
BERT: Bidirectional encoder representations from transformers
GNN: Graph neural networks
BCE: Binary cross-entropy
CNN: Convolutional neural networks
RNN: Recurrent neural network
MLP: Multi-layer perceptron
LSTM: Long short term memory
TF–IDF: Term frequency–inverse document frequency
ROC: Receiver operating characteristic
AUC: Area under the curve

## References

[1] Wang L, et al. Symptom effects and central mechanism of acupuncture in patients with functional gastrointestinal disorders: a systematic review based on fMRI studies. BMC Gastroenterol. 2024;24(1):47. 38267863 10.1186/s12876-024-03124-y PMC10809475

[2] Niazi S, et al. Psychosocial predictors of gastrointestinal symptomology in adults. In: Handbook of the biology and pathology of mental disorders. Cham: Springer, 2024;1–20.

[3] Sundas A, et al. Psychosocial quality–of–life correlates in functional gastrointestinal disorders. Rev Gastroenterol Méx (Engl Ed). 2024;89(1):11–8. 35810093 10.1016/j.rgmxen.2022.04.005

[4] Billey A, et al. The bidirectional relationship between sleep disturbance and functional dyspepsia: a systematic review to understand mechanisms and implications on management. Cureus. 2024;16(8):e66098. 39229406 10.7759/cureus.66098 PMC11370990

[5] Wang D, et al. Effects of acupuncture and moxibustion on ulcerative colitis: an overview of systematic reviews. Heliyon. 2024;10(6):e27524. 38510004 10.1016/j.heliyon.2024.e27524 PMC10951544

[6] Guo Y, Wei W, Chen JD. Effects and mechanisms of acupuncture and electroacupuncture for functional dyspepsia: a systematic review. World J Gastroenterol. 2020;26(19):2440–57. 32476804 10.3748/wjg.v26.i19.2440 PMC7243644

[7] The Four Diagnostic Methods of TCM [中医四诊 (望闻问切)]. In: Contextual dictionary of Chinese cultural knowledge. Singapore:Springer, 2025;942–944

[8] Liu R, et al. Traditional chinese medicine for functional gastrointestinal disorders and inflammatory bowel disease: narrative review of the evidence and potential mechanisms involving the brain–gut axis. Front Pharmacol. 2024;15:1444922. 39355776 10.3389/fphar.2024.1444922 PMC11443704

[9] Wang L, et al. GastroTCM: a large language model assistant for gastroenterology in traditional Chinese medicine. Chin Med. 2026;21(1):46. 41566401 10.1186/s13020-025-01295-8 PMC12825222

[10] Lin W, et al. Utilizing hierarchical efficacy regions of the human brain for treatment prediction through information fusion. Inf Fusion. 2026;129:103993.

[11] Zhang H, et al. Artificial intelligence boosting multi–dimensional information fusion: data collection, processing and modeling for food quality and safety assessment. Trends Food Sci Technol. 2025;163:105138.

[12] Li Y, et al. AcuKG: a comprehensive knowledge graph for medical acupuncture. J Am Med Inform Assoc: JAMIA. 2026;33(2):359–70. 41124298 10.1093/jamia/ocaf179 PMC12844574

[13] Zeng X, et al. LDComKG: an LLM–powered dual–enhanced framework for community–aware knowledge graph completion in traditional Chinese medicine. Health Inf Sci Syst. 2026;14(1):41. 41725781 10.1007/s13755-026-00429-y PMC12917067

[14] Huang X, et al. Integration of traditional Chinese medicine and machine learning: opportunities, obstacles, and implications for future of healthcare. J Integr Med. 2026;S2095–4964(26):21–8. 10.1016/j.joim.2026.02.004 41765716

[15] Wang J, et al. Multi–aspect co–attentional collaborative filtering for extreme multi–label text classification. Knowledge-Based Syst. 2023;260:110110.

[16] John JS, et al. Categorizing mental stress: a consistency–focused benchmarking of ML and DL models for multi–label, multi–class classification via taxonomy–driven NLP techniques. Nat Lang Process J. 2025;11:100162.

[17] Boonyarat P, et al. GNN–enabled max–min fair beamforming. Inf Process Manage. 2024;60(2):103192.

[18] Sun Y, et al. GTC: gnn–transformer co–contrastive learning for self–supervised heterogeneous graph representation. Neural Netw. 2025;181:106645. 39395234 10.1016/j.neunet.2024.106645

[19] Li H, et al. Acupoints compatibility rules of acupuncture for functional gastrointestinal disorders based on data mining technology: a systematic review. Iran J Public HEALTH. 2025;54(8):1591–607. 41069981 10.18502/ijph.v54i8.19568 PMC12507127

[20] Dong X, et al. Cross–domain neural collaborative filtering for personalized herbal prescription recommendation. Chin Med. 2026;21(1):57. 41606625 10.1186/s13020-025-01294-9 PMC12853632

[21] Han X, et al. Calculating the similarity between prescriptions to find their new indications based on graph neural network. Chin Med. 2024;19(1):124. 39261848 10.1186/s13020-024-00994-y PMC11391787

[22] Luo J, Yuan Y, Xu S. Improving GBDT performance on imbalanced datasets: An empirical study of class–balanced loss functions. Neurocomputing. 2025;634:129896.

[23] Kolbinger FR, Kather JN. Adaptive validation strategies for real–world clinical artificial intelligence. Nat Comput Sci. 2025;5(11):981–6. 10.1038/s43588-025-00901-x 41249673

[24] Wang F, et al. Knowledge structure of lumbago’s symptoms and employed acupoints in book AB classic of acupuncture and moxibustion and compendium of acupuncture and moxibustion based on weka preprocessing association rules. Bioinformatics. 2020;45(1):74–6. 10.13702/j.1000-0607.1803016 32144913

[25] Black CJ, et al. Functional gastrointestinal disorders: advances in understanding and management. Lancet. 2020;396(10263):1664–74. 33049221 10.1016/S0140-6736(20)32115-2

[26] Song M, et al. Status quo, problem, and prospect for traditional Chinese medicine international terminology standards. Guidel Stand Chin Med. 2025;3(1):1.

[27] Jia Q, et al. Traditional Chinese medicine symptom normalization approach leveraging hierarchical semantic information and text matching with attention mechanism. J Biomed Inf. 2021;116:103718. 10.1016/j.jbi.2021.103718 33631381

[28] WHO international standard terminologies on traditional chinese medicine. Accessed 15 Feb 2026. https://www.who.int/publications/i/item/9789240042322

[29] The fundamentals of acupuncture|nigel ching. Oxfordbookstore. Accessed February 17, 2026. https://oxfordbookstore.com/products/the-fundamentals-of-acupuncture

[30] He JZ, et al. Attention-based map encoding for learning generalized legged locomotion. Sci Robot. 2025;10(106):eadv3604. 10.1126/scirobotics.adv3604 40864729

[31] Wu X, et al. Repurposing loratadine to reverse colistin resistance in klebsiella pneumoniae through targeting lipid a modification. Emerg Microbes Infect. 2026;15(1):2623697. 41678146 10.1080/22221751.2026.2623697 PMC12903943

[32] Jin Z, et al. ACI-GNN: Lightweight All-Channel Interaction Graph Neural Network for Multi-Sensor Coal-Rock Cutting Recognition. Sensors. 2025. 10.3390/s25226820 PMC12655992 41305028

[33] Wang X, et al. mRSubLoc: a novel multi–label learning framework integrating RNA large language model for mRNA subcellular localization. IEEE J Biomed Health Inform. 2026;30(2):1821–9. 40694461 10.1109/JBHI.2025.3591454

[34] Jasodanand VH, et al. AI-driven fusion of multimodal data for Alzheimer’s disease biomarker assessment. Nat Commun. 2025;16(1):7407. 40789853 10.1038/s41467-025-62590-4 PMC12339743

[35] Wu JL, et al. Guideline-driven clinical decision support for colonoscopy patients using the hierarchical multi-label deep learning method. Chin Med J. 2025;138(20):2631-2639. 40405345 10.1097/CM9.0000000000003469 PMC12537133

[36] Abdeahad Y, et al. Accurate and robust ambiguity detection in software requirements documents using a GloVe-BiLSTM deep learning framework with data augmentation. Sci Rep. 2026;16(1):18996. 10.1038/s41598-026-48700-2 PMC13276063 42031899

[37] Pei Z, et al. A study on quantitative analysis methods for mixed solution concentrations based on deep learning. Spectrochim Acta A Mol Biomol Spectrosc. 2026;359:127894. 42035660 10.1016/j.saa.2026.127894

[38] Zhao L, et al. Conjoint feature representation of GO and protein sequence for PPI prediction based on an inception RNN attention network. Mol Ther Nucleic Acids. 2020;22:198–208. 33230427 10.1016/j.omtn.2020.08.025 PMC7515979

[39] Yuan D, et al. Qwen TextCNN and BERT models for enhanced multilabel news classification in mobile apps. Sci Rep. 2025;15(1):43787. 41398002 10.1038/s41598-025-27497-6 PMC12706044

[40] Wang M, et al. Emerging infectious disease surveillance using a hierarchical diagnosis model and the Knox algorithm. Sci Rep. 2023;13(1):19836. 37963966 10.1038/s41598-023-47010-1 PMC10645817

[41] Aktas A, et al. Advanced multi–level ensemble learning approaches for comprehensive sperm morphology assessment. Diagnostics. 2025;15(12):1564. 40564884 10.3390/diagnostics15121564 PMC12192502

[42] Tian Y, et al. Trace–level detection of free polycyclic aromatic hydrocarbons based on magnetic driving and deep learning–assisted recognition. Spectrochim Acta A Mol Biomol Spectrosc. 2026;352:127531. 41621160 10.1016/j.saa.2026.127531

[43] Wang XF, et al. a dynamic multi-scale hypergraph learning framework driven by features and structures for ceRNA–disease association prediction. IEEE J Biomed Health Inform. 2026;30(3):2252–62. 40853813 10.1109/JBHI.2025.3602670

[44] Yang YW, et al. Automated flow and local LLM–driven clinical context engineering: precision colorectal cancer recurrence registry. Int J Med Inf. 2026;213:106383. 10.1016/j.ijmedinf.2026.106383 41849918

[45] Li M, et al. Automated identification of nursing diagnoses and interventions from nursing records using a retrieval–augmented large language model approach: quantitative study. J Med Internet Res. 2026;28:e89850. 42054561 10.2196/89850 PMC13128066

[46] Krishnan UCA, et al. Generative AI in disability–inclusive learning: a bibliometric and systematic literature analysis. Disabil Rehabil, Assist Technol. 2026;1–30 10.1080/17483107.2026.2653073 41973863

[47] Zhou HX, et al. NutriRAG: unleashing the power of large language models for food identification and classification through retrieval methods. J Am Med Inform Assoc. 2026;33(4):802-811. 10.1093/jamia/ocag003 PMC13005737 41617202

[48] Yang B, Song P. Enhanced anchor contrastive multi–view representations learning network for clustering. Neural Netw: Off J Int Neural Netw Soc. 2026;195:108233. 10.1016/j.neunet.2025.108233 41151526

[49] Chen F, et al. MAGED: multimodal attentive graph learning with gene expression dynamics on knowledge graphs for TCM target prediction. J Ethnopharmacol. 2026;361:121218. 41547391 10.1016/j.jep.2026.121218

[50] Abugabah A, et al. An intelligent healthcare system for rare disease diagnosis utilizing electronic health records based on a knowledge–guided multimodal transformer framework. Biodata Min. 2025;18(1):70. 41057892 10.1186/s13040-025-00487-0 PMC12505588

[51] Liu R, et al. HetGAT–LMI: interpretable heterogeneous graph attention method for predicting lncRNA–miRNA interactions. J Chem Inf Model. 2026;66(1):796–807. 41432189 10.1021/acs.jcim.5c02664

[52] Guo D, et al. Predicting complications and mortality in myocardial infarction patients using a graph neural network model. Sci Rep. 2026;16(1):5886. 41565987 10.1038/s41598-026-36798-3 PMC12894713

[53] Ye R, et al. Acupuncture improves anxiety and depression in patients with polycystic ovary syndrome: a systematic evaluation and meta–analysis. Front Med. 2026;13:1738629. 10.3389/fmed.2026.1738629 PMC12868136 41647523

[54] Ke HJ, et al. Unsupervised deep frequency–channel attention factorization to non–linear feature extraction: a case study of identification and functional connectivity interpretation of Parkinson’s disease. Expert Syst Appl. 2024;243:122853.

[55] Wang FQ, et al. Deep wavelet temporal-frequency attention for nonlinear fMRI factorization in ASD. Pattern Recognit. 2025;165:111543.

[56] Wagner M, et al. Lost in homogenisation: Navigating the challenges of predicting ideal behaviour in inhomogeneous porous structures. Int J Solids Struct. 2025;320:113522.

[57] Ke HJ, et al. Deep factor learning for accurate brain neuroimaging data analysis on discrimination for structural MRI and functional MRI. IEEE/ACM Trans Comput Biol Bioinformatics. 2024;21(4):582–95. 10.1109/TCBB.2023.3252577 37028037

[58] Ke HJ, et al. ADHD identification and its interpretation of functional connectivity using deep self–attention factorization. Knowledge-Based Syst. 2022;250:109082.

[59] Wang FQ, Ke H, Tang Y. Fusion of generative adversarial networks and non–negative tensor decomposition for depression fMRI data analysis. Inf Process Manage. 2025;62(2):103961.

[60] Mansoor I, et al. Reasoning with large language models in medicine: a systematic review of techniques, challenges and clinical integration. Health Inf Sci Syst. 2026;14(1):6. 41323158 10.1007/s13755-025-00403-0 PMC12657685

